# The AMPA receptor antagonist perampanel suppresses epileptic activity in human focal cortical dysplasia

**DOI:** 10.1002/epi4.12549

**Published:** 2022-05-11

**Authors:** Anderson Brito da Silva, Jane Pennifold, Ben Henley, Koustav Chatterjee, David Bateman, Roger W. Whittaker, Abhijit Joshi, Hrishikesh Kumar, Claire Nicholson, Mark R. Baker, Stuart D. Greenhill, Richard Walsh, Stefano Seri, Roland S. G. Jones, Gavin L. Woodhall, Mark O. Cunningham

**Affiliations:** ^1^ Institute of Neuroscience The Medical School Newcastle University Newcastle upon Tyne UK; ^2^ Ministry of Education of Brazil CAPES Foundation Brasília – DF Brazil; ^3^ Aston Brain Centre School of Life and Health Sciences Aston University Birmingham UK; ^4^ Institute of Neurosciences Kolkata Kolkata India; ^5^ Department of Neurology Sunderland Royal Hospital Sunderland UK; ^6^ Department of Clinical Neurophysiology Royal Victoria Infirmary The Newcastle upon Tyne Hospitals NHS Foundation Trust Newcastle upon Tyne UK; ^7^ Department of Neuropathology Royal Victoria Infirmary The Newcastle upon Tyne Hospitals NHS Foundation Trust Newcastle upon Tyne UK; ^8^ Department of Neurosurgery Royal Victoria Infirmary The Newcastle upon Tyne Hospitals NHS Foundation Trust Newcastle upon Tyne UK; ^9^ Children's Epilepsy Surgery Service Birmingham Women's and Children's NHS Foundation Trust Birmingham UK; ^10^ Department of Pharmacology and Pharmacy University of Bath Bath UK; ^11^ Discipline of Physiology School of Medicine Trinity College Dublin Dublin 2 Ireland

**Keywords:** AMPA, epilepsy, focal cortical dysplasia, glutamate, perampanel

## Abstract

Focal cortical dysplasia (FCD) is one of the most common malformations causing refractory epilepsy. Dysregulation of glutamatergic systems plays a critical role in the hyperexcitability of dysplastic neurons in FCD lesions. The pharmacoresistant nature of epilepsy associated with FCD may be due to a lack of well‐tolerated and precise antiepileptic drugs that can target glutamate receptors. Here, for the first time in human FCD brain slices, we show that the established, noncompetitive α‐amino‐3‐hydroxy‐5‐methyl‐4‐isoxazolepropionic acid (AMPA) receptor antagonist, perampanel has potent antiepileptic action. Moreover, we demonstrate that this effect is due to a reduction in burst firing behavior in human FCD microcircuits. These data support a potential role for the treatment of refractory epilepsy associated with FCD in human patients.

**KEY POINTS**

- FCD IIb lesions are frequently pharmacoresistant and require alternative therapeutic options
- *Ex vivo* electrophysiological studies in human FCD IIb brain slices obtained from neurosurgery reveal that a clinically approved AMPA receptor antagonist can inhibit seizure activity at therapeutically relevant concentrations
- These findings suggest that pharmacological approaches that antagonise AMPA receptors may be worth exploring in the treatment of seizures associated with FCD IIb

## 1 INTRODUCTION

Focal cortical dysplasia type IIb (FCD IIb) lesions are notoriously epileptogenic and associated with drug‐refractory epilepsy. From a histological point of view, FCD IIb presents with cytoarchitectural abnormalities of the neocortex, often with the presence of balloon cells, dysmorphic neurons, and hypomyelinated white matter. Due to the propensity for pharmacoresistance in FCD IIb, a significant proportion of patients will undergo surgical resection of the lesion in order to control seizures. The ability to conduct a “complete” resection can be complicated by a number of factors. Crucially, due to the fact that FCD IIb lesions frequently occur close to eloquent cortex and important fiber tracts, it is not always possible to conduct a full resection as this may lead to postsurgical neurological deficits. Therefore, alternative therapeutic approaches for epilepsy arising from this particular type of lesion are required.

There is evidence to suggest a role for glutamate in the refractory nature of FCD IIb. Increased glutamatergic input and altered expression/function of N‐methyl‐D‐aspartate receptors has been demonstrated in surgical samples from patients with FCD IIb. An abundance of vesicular glutamate transporter 1 (VGLUT1)–positive synapses on dysmorphic neurons in the epileptogenic focus of a FCD IIb^1^ is likely to lead to increased cortico‐cortical excitatory input to dysplastic regions. Moreover, there have also been reports of increased glutamate signal in some patients with cortical developmental malformations as detected by magnetic resonance imaging.^2^ Indeed, seizure generation in human microcircuits is dependent upon the emergence of population glutamatergic activity.^3^

Given the refractory nature of epilepsy caused by FCD IIb lesions and the hypothesis that glutamate may be elevated in and around FCD IIb lesions, we have aimed to examine the impact of an antiepileptic drug that acts to noncompetitively antagonize α‐amino‐3‐hydroxy‐5‐methyl‐4‐isoxazolepropionic acid (AMPA) receptors. A number of compounds have been demonstrated to antagonize the AMPA receptor. These include experimental compounds such as 6‐nitro‐2,3‐dioxo‐1,4‐dihydrobenzo[f]quinoxaline‐7‐sulfonamide (NBQX) and LY293558. A number of AMPA receptor antagonists have been employed in clinical trials (eg, talampanel), but only one has been approved for clinical use, This drug, perampanel (Fycompa), is capable of maintaining the closure of AMPA receptors even in the presence of elevated glutamate levels. Perampanel is currently licensed for the adjunctive treatment of partial‐onset seizures with or without generalized seizures in children (≥12 years old) and adults and has also been approved for use in genetic generalized epilepsy.^4^ Previous ex vivo studies have examined the impact of perampanel on epileptiform activity induced by the addition of picrotoxin^5^ and altered extracellular potassium (K^+^) and magnesium (Mg^2+^) levels in the artificial cerebrospinal fluid (ACSF) in resected hippocampal slices from a single juvenile patient with refractory temporal lobe epilepsy.^6^ A recent paper has demonstrated the ability of perampanel to suppress spontaneous epileptiform activity via a selective inhibitory effect on excitatory posy‐synaptic currents.^7^ Here, for the first time, we demonstrate the efficacy of perampanel on ex vivo human ictal activity arising from a neocortical developmental abnormality associated with intractable epilepsy.

## 2 METHODS

The electrophysiological data obtained from slice studies were derived from three patients with medically intractable epilepsy who were undergoing elective neurosurgical tissue resection for the removal of a suspected FCD. Before surgery, all patients gave their informed consent for the use of the resected brain tissue for scientific studies. This study was approved by the County Durham & Tees Valley 1 Local Research Ethics Committee (06/Q1003/51) (date of review 03/07/06) and had clinical governance approved by the Newcastle upon Tyne Hospitals NHS Trust (CM/PB/3707). Studies at Aston were approved by the Black Country Local Ethics Committee (10/H1202/23; 04/30/10), the Aston University ethics committee (Project 308 cellular studies in epilepsy), and through the Research and Development Department at Birmingham Children's Hospital (IRAS ID12287).

### 2.1 In vitro human neocortex recordings

Briefly, human cortical samples were derived from material removed as part of surgical treatment of medically intractable cortical epilepsy from the left parietal lobe, left frontal lobe, and right temporal lobe regions with written informed consent of the patients (N = 3). Slice preparation and extracellular recording were conducted using methods as previously described.^8^ The time between resection and slice preparation was <5 minutes. Multielectrode array (MEA) recordings were conducted using Buzsaki‐style probes (64‐electrode; NeuroNexus Technologies) connected to an Intan RHD2000‐series amplifier system (Intan Technologies). Signals were amplified and digitized (20 kHz) using the Intan system and downsampled to 2 kHz for offline analysis of the extracellular local field potential (LFP) using MATLAB (MathWorks).

### 2.2 Extracellular data and statistical analysis

For LFP recordings, power spectrum analysis was calculated by integrating the root mean square value of the signal in frequency bands from 1 to 1000 Hz in sequential 10‐minute time windows in the baseline state and following the application of perampanel. Power values were expressed as raw values and a paired t test was applied to examine statistical significance between baseline and following drug application. Multiunit activity (MUA) was extracted from each channel of the MEA using a MATLAB (MathWorks, Natick, Maine) script available in the wave_clus toolbox.^9^ The signal was band‐pass filtered between 300 Hz and 3 kHz, and a threshold was calculated through an estimation of the standard deviation of the background noise ($\delta_{n}$), as shown below.

$$\delta_{n}=\mathrm{median}\left(\frac{\left|x\right|}{0.6745}\right)$$

Negative deflections in the filtered signal that were more than five times this estimated background noise were selected. An mxn matrix was created with the waveforms, where *n* is the total number of detections and *m* is the number of samples for each waveform. The spikes were aligned in the point of maximum negative deflection after an upsampling to 120 kHz through cubic spline interpolation. Finally, each waveform was visually inspected to exclude events of clear artifactual origin. For the analysis of high‐frequency oscillations (HFOs) from the MEA recordings, an “in‐house” HFO detection algorithm was applied. A nonparametric paired test (Wilcoxon) was used to calculate statistical significance. To quantify the difference in parameters between the conditions, a percentage of difference was calculated for each channel using the equation below, where $X_{\mathrm{PER}}$ and $X_{\mathrm{baseline}}$ are the parameter X (HFO, MUA, or Power) in the perampanel‐treated and baseline conditions, respectively.

$$\mathrm{Difference}\;\left(\%\right)=100*\frac{X_{\mathrm{PER}}‐X_{\mathrm{baseline}}}{X_{\mathrm{Baseline}}}$$

All statistical calculations were performed with GraphPad Prism (La Jolla, California).

## 3 RESULTS

**FIGURE 1:**
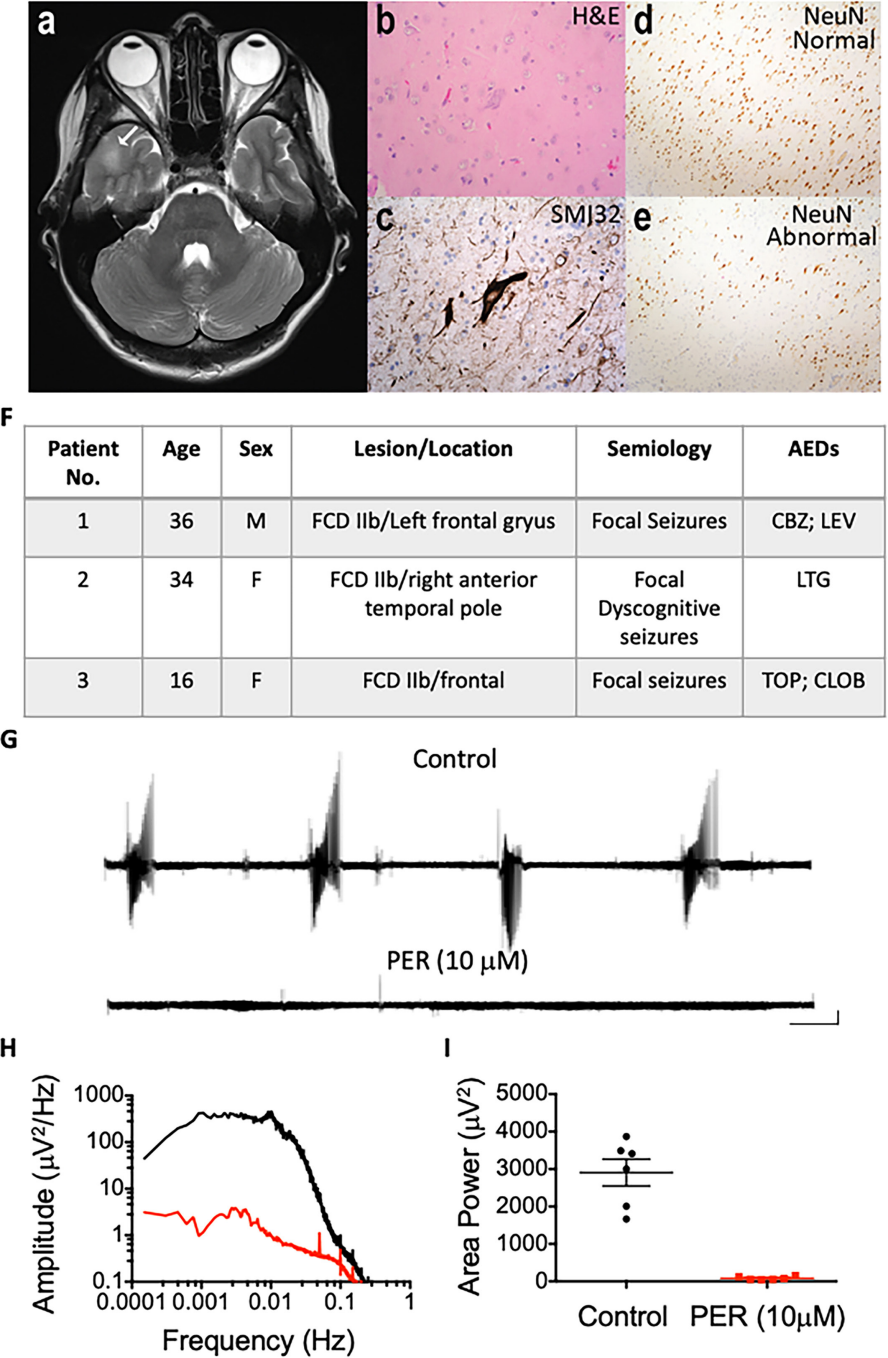
Suppression of seizure activity by perampanel in human FCD brain slices. (A) Axial T2 MR image (from Patient 2) through the temporal poles illustrating the location (white arrow) of an FCD lesion in the right lateral anterior temporal pole; (B) H&E‐stained sections showing dysmorphic neurons; (C) immunohistochemistry for nonphosphorylated neurofilament protein (SMI32) showing aberrant NFP accumulation in dysmorphic neurons; (D) NeuN immunohistochemistry illustrating regions with better preserved cortical structure compared with (E) which shows regions with abnormal architecture and neuronal depletion; (F) patient age, sex, FCD IIb lesion and location, semiology, and AEDs; (G) raw LFP traces illustrating ictal discharges recorded in 0.25 mM Mg^2+^ and 8 mM K^+^ in the absence (top) and presence of perampanel (bottom); (H) power spectra generated from example traces in the absence (black) and presence of perampanel (red); (I) area power (1‐1000 Hz) values for all experiments demonstrating the ability of perampanel to suppress epileptiform activity in all samples tested. Scale bars represent 0.5 mV and 1 min. AED, antiepileptic drug; CBZ, carbamazepine; CLOB, clobazam; FCD, focal cortical dysplasia; H&E, hemotoxylin and eosin; LEV, levetiracetam; LFP, local field potential; LTG, lamotrigine; MRI, magnetic resonance imaging; NeuN, neuronal nuclear antigen; NFP, neurofilament protein; PER, perampanel; TOP, topiramate

Similar to previous studies, spontaneous epileptic activity was not observed in slices prepared from resected tissue from patients with FCD.^10^, ^11^ In order to elicit epileptiform activity, slices were perfused with modified ACSF containing reduced Mg^2+^ (0.25 mM) and elevated K^+^ (8 mM). Previous studies have demonstrated that the use of this modified ACSF (mACSF) is known to induce ictal events in brain slices obtained from patients with a history of epilepsy.^3^ Preliminary data from our group have shown that application of this mACSF to nonepileptic comparison tissue does not elicit epileptic activity (Cunningham et al, unpublished data). These findings suggest that this particular type of human epileptic tissue demonstrates a reduced threshold for ictal behavior due to alterations in cellular and network excitability.^10^ Following application of the mACSF, recurring ictal events emerged (Figure 1G), and once established, a mean (± standard error of the mean) area power value of 2807 ± 418.1 µV^2^ was observed across all samples examined (N = 6). Upon application of perampanel (10 µM), ictal events were abolished and power spectral analysis revealed that the area power was significantly reduced to 114.6 ± 30.1 µV^2^ (paired student t test, *P* < .05; Figure 1H,I).

**FIGURE 2:**
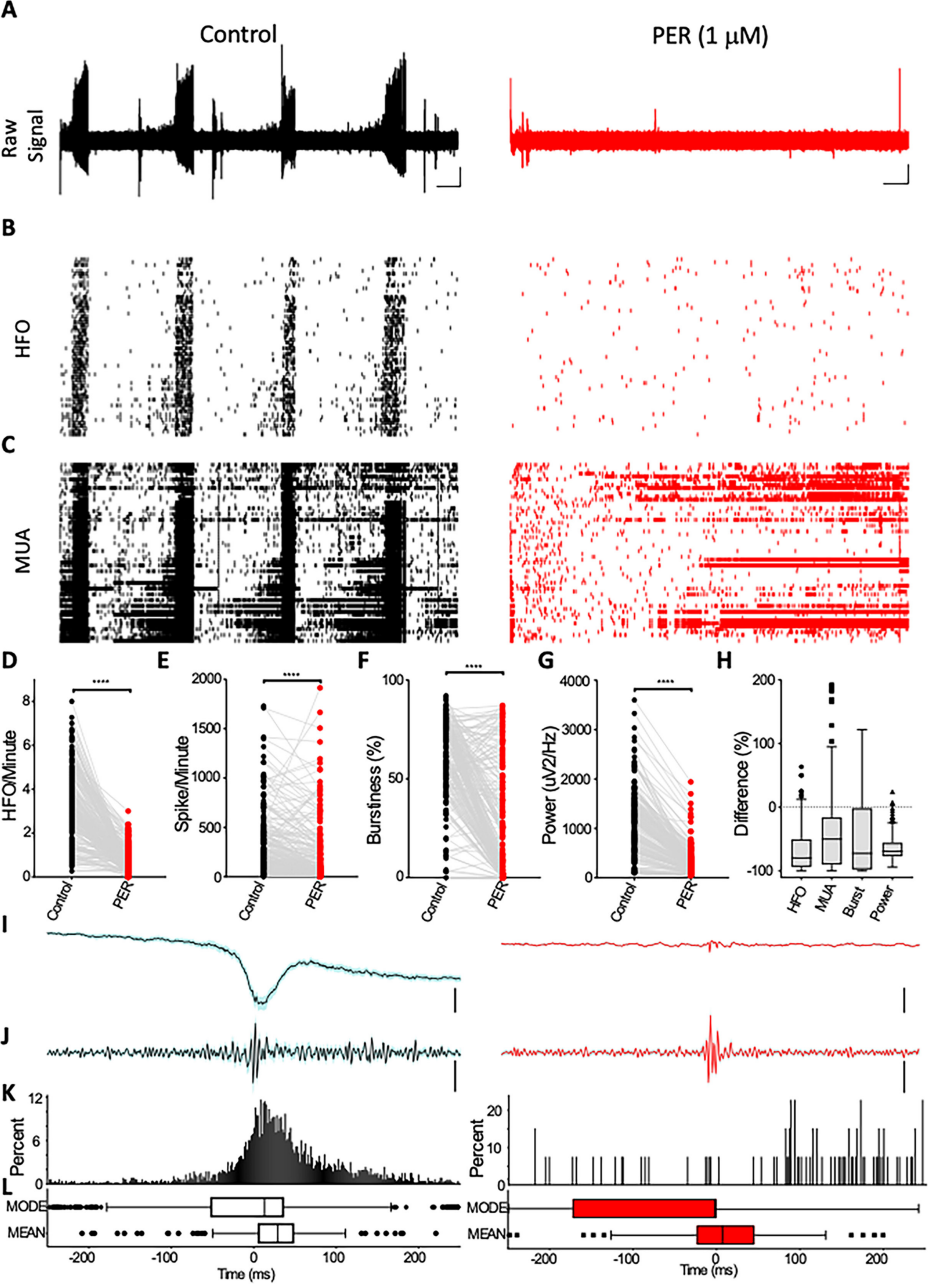
Impact of therapeutically relevant concentration of perampanel on epileptic network dynamics. MEA recordings of ictal discharges recorded in 0.25 mM Mg^2+^ and 8 mM K^+^ showing (A) raw LFP, (B) HFO, and (C) MUA rastergram behavior in baseline (left) conditions and in the presence of perampanel (1 µM) (right). Graphs illustrate the effect of perampanel on (D) HFO, (E) MUA, (F) burstiness, and (G) LFP for all channels analyzed, and (H) shows the impact of perampanel for each of these variables represented as percentage change; all parameters were significantly reduced when washed with perampanel (paired Wilcoxon test: ****P < .0001). Correlation between HFO and MUA showing (I) the average (black line) and standard error (grey shadow) of raw signal segments containing HFO from one example channel; (J) average of the same segments in (I) filtered between 60 and 600 Hz; (K) cross‐correlograms of the time difference between MUA and HFO for the same channel displayed in (I) and (B); for the baseline condition, n = 153 HFO and for the treated condition, n = 47 HFO. (L) Boxplot of the mean and mode of all cross‐correlograms (as displayed in K) from all channels (n = 250 channels). Scale bars for (A) represent 0.1 mV and 1 min and for (I) and (J) represent 40 and 5 µV, respectively. HFO, high‐frequency oscillations; LFP, local field potential; MEA, multielectrode array; MUA, multiunit activity; PER, perampanel

In patients with epilepsy, plasma concentrations of perampanel have been reported to vary between 1.06 and 3.26 µM.^12^ In order to examine the effect of a therapeutic concentration of the drug on FCD epileptic activity, we next tested the drug at a lower concentration (1 µM). In these studies, we used MEA technology in an attempt to capture the activity of single neurons in tandem with LFP epileptic activity and, therefore, understand the impact of perampanel on collective neuronal firing behavior. As was observed in the glass microelectrode studies (Figure 1G‐I), the addition of perampanel (1 µM) significantly reduced the power of ictal LFP activity (Figure 2A) (control – median: 872.6 μV^2^/Hz, interquartile range [IQR]: 384.5‐1434 vs perampanel – median: 214.3 μV^2^/Hz, IQR: 110‐352.8; reduction of 69.1%, IQR: −76.96 to −55.59) recorded using MEAs (Figure 2D). Recordings with MEAs revealed bursts of single‐unit action potentials occurring concurrently with the ictal LFP event (Figure 2C). The addition of perampanel significantly reduced the spike count (control – median: 201.3 spikes/minute, IQR: 72.7‐439.7 vs perampanel – median: 65.9 spikes/minute, IQR: 4.28‐274.6; reduction of 49.96%, IQR: −89.97 to −15.95), and bursting behavior (control – median: 61.9%, IQR: 46.3‐77.5 vs perampanel – median: 11.9%, IQR: 0.02‐51.3%; reduction of 72.3%, IQR −97.6 to −1.5 (Figure 2E‐G)). High‐frequency oscillations (HFOs), a hallmark of epileptogenic networks, were also significantly reduced by the application of the drug (control – median: 3.06 HFO/minute, IQR: 2.02‐4.38 vs perampanel – median: 0.53 HFO/minute, IQR: 0.27‐1.05; reduction of 79.51%, IQR: −93.48 to −50.19 (Figure 2D)). Perampanel was also observed to significantly reduce the degree of cross correlation between multiunit activity and HFOs (Figure 2I‐L).

## 4 DISCUSSION

These electrophysiological and pharmacological data demonstrate that the use of perampanel is effective in reducing seizure‐like activity in neocortical slices resected from human patients with FCD, suggesting potential benefit for the use of this drug in treating pharmacoresistant epilepsy in this patient group.

Mechanistically, perampanel has been demonstrated to be a potent noncompetitive inhibitor of AMPA receptors.^13^ Studies conducted in rodent hippocampal slices have demonstrated the direct inhibition of AMPA‐receptor–mediated currents,^14^ and AMPA‐mediated synaptic transmission.^15^ AMPA receptors are crucial for generation, synchronization, and spread of epileptic discharges.^16^ In cortical tissue removed from patients with partial‐onset epilepsy, AMPA receptor density is increased,^17^ and the sensitivity of the receptor to glutamate enhanced by altered RNA editing.^18^

Previous work has suggested that AMPA receptors are a potential target for therapeutic intervention in patients with epilepsy associated with FCD. In one study examining mRNA expression, AMPA receptor subunit transcripts (GluR4) were increased in dysplastic neurons.^19^ The mammalian target of rapamycin (mTOR) pathway has emerged as a primary pathogenic mechanism underlying cortical lesions such as FCD IIb. Preclinical and clinical studies have demonstrated the effectiveness of mTOR inhibitors in treating FCD, although the mechanism remains unclear. Studies in neuronal cultures have demonstrated that mTOR inhibitors significantly reduced the surface expression of AMPA receptors on cortical neurons,^20^ thus supporting a potential role for AMPA receptors in epileptic FCD networks. Pharmacological blockade of AMPA receptors constitutes a more readily available therapeutic option. Extracellular recordings in human epileptic tissue have previously demonstrated that ictal events are sensitive to AMPA receptor antagonism using a competitive blocker (6‐nitro‐2,3‐dioxo‐1,4‐dihydrobenzo[f]quinoxaline‐7‐sulfonamide; NBQX).^3^ Whole‐cell patch clamp recordings in human FCD brain slices have shown that excitatory postsynaptic currents mediated via AMPA receptors where abolished by perampanel, whereas inhibitory events mediated via GABA_A_ receptor were relatively unaltered.^7^ This differential effect of perampanel is likely to underlie the profound antiseizure effect we report in our current study. In the present study, concentrations similar to those used by Wright et al (2020)^7^ (10 μM) produced a complete suppression of ictal activity. In addition, we have showed that the suppression of ictal LFP activity and associated HFOs is maintained by concentrations of perampanel likely to be observed in plasma concentrations in human patients (c. 1 μmol/L).^12^ The MEA recordings in human tissue revealed that the pathological bursting activity of neurons (which is coincidental with ictal discharges) is strongly inhibited. Interestingly, in the presence of the lower concentration of perampanel, a large proportion of neurons are still active. This finding supports the notion that AMPA‐mediated synaptic conductances are critical for bursting behavior that drives ictal activity^16^ and perampanel is capable of blocking these specific synaptic conductances and thus limiting associated epileptic activity (Figure 2H) in FCD neuronal microcircuits.

Additional work is required to understand the role of AMPA receptors in FCD and, in particular, the therapeutic potential of perampanel in this patient group. Our ex vivo human tissue findings are, to a degree, corroborated clinically by a retrospective analysis study^21^ showing seizure suppression with perampanel in adolescent patients with FCD. It remains to be seen if the results presented here are translationally robust in a clinical setting. A recent observational multicenter study has shown efficacy and safety for perampanel as an adjunctive therapy in a drug‐resistant focal epilepsy patient cohort that had a significant number of participants with focal cortical dysplasias.^22^ In that respect, a randomized, controlled trial examining the clinical efficacy of perampanel in patients with FCD is warranted.

## CONFLICT OF INTEREST

None of the authors has any conflict of interest to disclose.

We confirm that we have read the Journal's position on issues involved in ethical publication and affirm that this report is consistent with those guidelines.

## AUTHOR CONTRIBUTIONS

MRB, SS, RSGJ, GLW, and MOC contributed to the conception and design of the study; ABdS, BH, SDG, RW, JP, KC, DB, RWW, AJ, HK, CN, MRB, GLW, and MOC contributed to the acquisition and analysis of data; and GLW, RSGJ, MRB, and MOC provided a substantial contribution to drafting the paper.
